# Interconnection morphology effects on the radio frequency response of carbon nanotube sponges

**DOI:** 10.3762/bjnano.17.23

**Published:** 2026-02-17

**Authors:** Manuela Scarselli, Javad Rezvani, Zeno Zuccari, Mattia Scagliotti, Simone Tocci

**Affiliations:** 1 Dipartimento di Fisica, Università di Roma Tor Vergata, Via della Ricerca Scientifica 1, 00133 Roma, Italy; 2 INFN-Sezione di Roma Tor Vergata, Via della Ricerca Scientifica 1, 00133 Roma, Italy; 3 University of Camerino, Dipartimento di Fisica, Via Madonna delle Carceri 9B, 62032 Camerino (MC), Italy; 4 INFN-Laboratori Nazionali di Frascati, Via Enrico Fermi 54, 00044 Frascati (Rm), Italy; 5 CNR-IMM Istituto per la Microelettronica e Microsistemi, Consiglio Nazionale delle Ricerche, Via del Fosso del Cavaliere 100, 00133 Roma, Italy

**Keywords:** carbon nanotube film, carbon nanotube sponge, chemical vapour deposition, monopole RF antenna

## Abstract

In this work, the high-frequency response of a multiwalled carbon nanotube (MWCNT) film grown on a silicon substrate is compared with that of MWCNT sponges (CNSs). Different from the CNT film, CNSs are a self-standing material that can operate in the absence of a supporting substrate, showing high flexibility, light weight, and mechanical robustness. We tested our synthesized CNSs as active material for the production of antennas working in the radio frequency (RF) range to determine whether CNT sponges present, in addition to practical advantages over CNT films, also an actual performance gain. The antenna built from CNSs shows an enhanced response gain compared with that of the MWCNT film, with both antennas having a maximum positioned around 4.8 GHz. After identifying the best CNT-based sample, the experiment focused on improving the CNS antenna’s response. In particular, we observed that the response of *S*_11_ = −22.6 dB around 4.8 GHz from the CNS antenna improved after a mild treatment with ethanol, reaching S_11_ = −32.6 dB measured after 10 min of waiting. This observed effect is studied in detail with scanning electron microscopy and Raman spectroscopy, which point to significant modifications of the CNS’s inner morphology after the treatment. Signal reception tests simulating real-world operation conditions were also carried out at two different distances to evaluate the practical application of the CNS as RF antennas. The ethanol treatment was also applied for these tests, and an increase in the response up to 45% was found for the two studied positions.

## Introduction

Most radio frequency (RF) applications rely on the use of conductors with low resistivity, especially metals, which, when applied in antennas, guarantee good radiation of electromagnetic (EM) waves into free space. Applications of wireless technology are constantly expanding regarding short-range connections, like wireless local area networks and Bluetooth, and wide-area coverage, like cellular networks (4G/5G) for mobile and satellite communication. This vast number of applications often requires that the active materials possess additional properties beyond good electrical conductivity, such as flexibility, lightness, and resistance to thermal and mechanical stress or chemically harsh conditions [1–2]. Novel alternative materials including conducting polymers [3], nanocarbon-based materials like carbon fibres [4], carbon nanotubes (CNTs), their composites [5–7], and more recently graphene [8–9] have been the focus of extensive research. Above materials fulfil the requirements of being lighter and more resistant to a broad range of environmental and harsh operating conditions, while maintaining sufficient electrical conductivity. Among them, CNTs [10–11] proved to be well-performing materials in electronics and optoelectronic applications. CNT-based antennas have been realized from single-walled CNTs (SWCNTs) [12–13] as well as multiwalled CNTs (MWCNTs) [14–17]. CNTs are characterized by slow wave propagation and high characteristic impedance, and their conductivity can vary as a function of dimensions (length and diameter), chirality, and purity [16]. This enables the design of resonance antennas with dimensions down to one fiftieth or less of conventional metallic resonance antennas [4]. In addition, the electrons can travel freely along the 1D length of the tubes but not in the transverse direction, so that the high-frequency skin effect usually observed in conductors is not appreciable. Interesting results have been found for shear-aligned CNT films, which showed a radiation efficiency of 94% at 10 and 14 GHz, comparable to copper antennas [18]. Threads made of twisted bundles of as-grown CNTs worked as dipole antennas resonating at 2.45 GHz [6]. A recent study on SWCNT antennas built on flexible substrates measured slight changes in the radiation efficiency gain from 80.0% for flat to 83.7% for bent configurations, demonstrating that CNTs are suitable for applications requiring non-standard shapes and surfaces [13].

MWCNTs possess larger physical dimensions compared to SWCNTs and have higher conductivity. Wang et al. [15], demonstrated that films of MWCNTs can operate as antennas when irradiated with light in the visible and near-UV regions. In particular, the maximum intensity of the reflected light in the visible range is obtained when the average length of the nanotubes composing the film is a half-integer multiple of the wavelength of the incident light (i.e., operation as simple dipole radio antennas). Similar to SWCNTs, MWCNTs can be doped or threaded/bundled to enhance conductivity, and it was found that, when the thickness of MWCNTs was increased from 0.5 to 5.0 μm, the gain changed from 14% up to 70% [19]. MWCNTs were also mixed with conductive polymers [20–21] or with metals [22], showing sizeable improvements in the response.

More recently, a novel material based on a 3D self-standing CNT assembly, often denoted as carbon nanotube sponge (CNS) [23], was applied as active medium for the fabrication of antennas [24]. The material consists of highly interconnected MWCNTs that are assembled during the synthesis process and confer the material its 3D shape. In addition, the assembly has interesting macroscopical properties like high porosity, low weight, good mechanical response, and good conductivity [25]. The properties of CNSs have been exploited in many different applications that include mechanical transducers [26], water filtration [27], and others [28].

In this paper, we tested our synthesized CNSs as active material for the production of antennas working in the RF range and compared them to a film of MWCNTs to determine whether CNT sponges offer, in addition to practical advantages over CNT films, also an actual performance gain.

We also found that the response in the RF range can be significantly improved after a mild treatment of the CNS in ethanol. The obtained results are discussed, correlating the EM measurements with morphological and structural analyses.

## Results and Discussion

**Figure 1:**
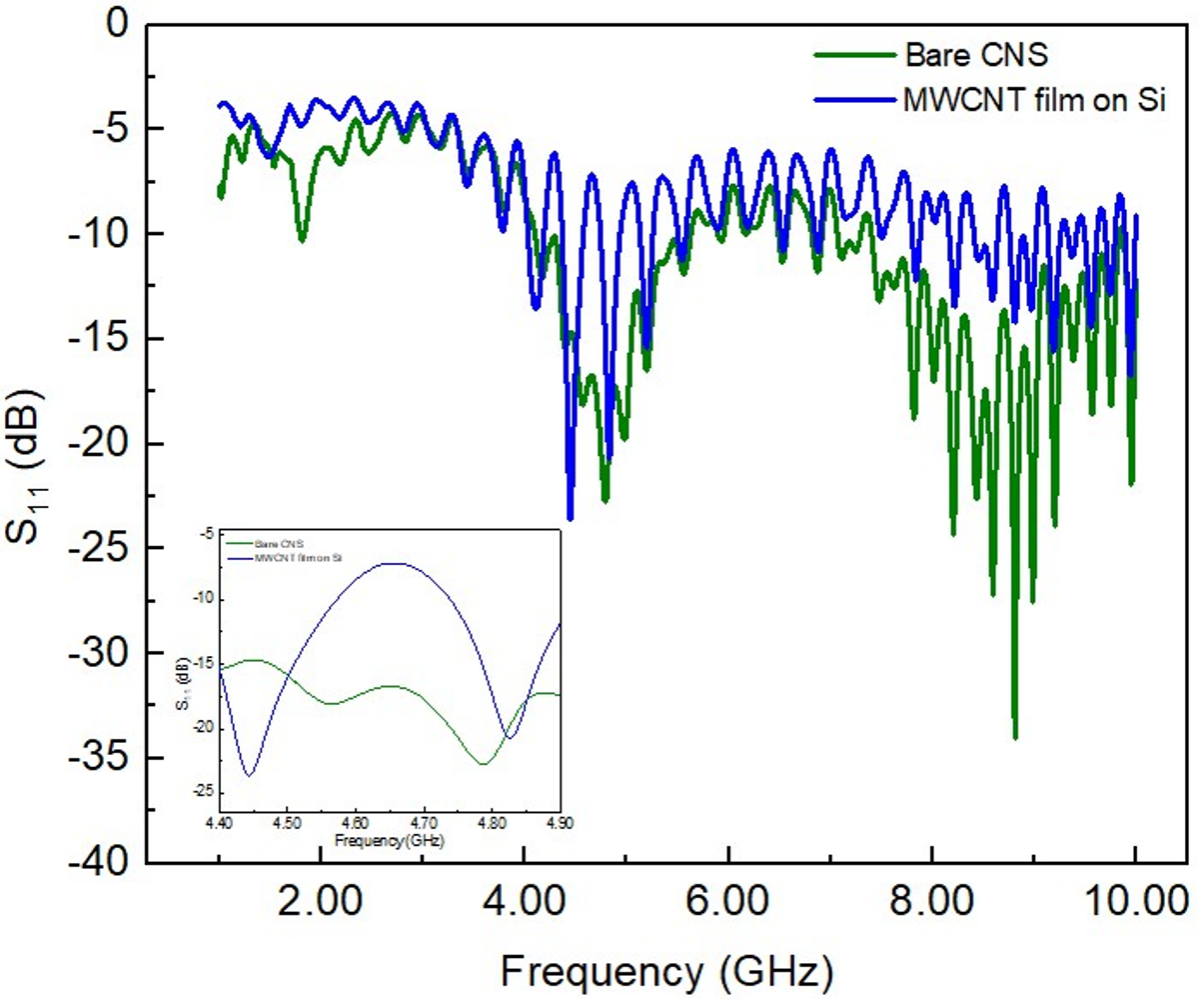
Measured *S*_11_ plot of the CNS sample (green line) and the MWCNT film grown on Si substrate (blue line). Inset: enlarged view of the same spectra in the region centred around 4.6 GHz.

We first studied the *S*_11_ parameter to get insight into the antenna’s impedance matching and resonant behaviour. We acquired the *S*_11_ signal from the as-grown CNS sample and the film of MWCNTs grown on a Si substrate as comparison (Figure 1). We observed that both samples have a resonance peak; for the CNT film, it is *S*_11_ = −23.0 dB at 4.40 GHz, and for the CNS sample, it is *S*_11_ = −22.6 dB at 4.78 GHz.

The result found for the MWCNT film is comparable to similar measurements reported in the literature, but located at a different frequency [17–19]. This observed discrepancy can be ascribed to differences in the synthesis parameters (e.g., temperature, precursors, solvents, and use of a substrate) and the choice of the substrate [16]. In our case, while the CNS sample is self-standing, the CNT film was grown on a silicon substrate, and the combination of a substrate and slight differences in the growth parameters explains the discrepancy in resonance peak position. The CNS sample also shows an additional resonance at 8.8 GHz with *S*_11_ = −34 dB, while the CNT film has no resonance around this frequency. The resonance around 4.7 GHz is the most interesting one because it matches many communications applications [1–2]. Therefore, we focused on the 4–5 GHz frequency range, disregarding the higher frequency signal since it is less stable and less applicable.

**Figure 2:**
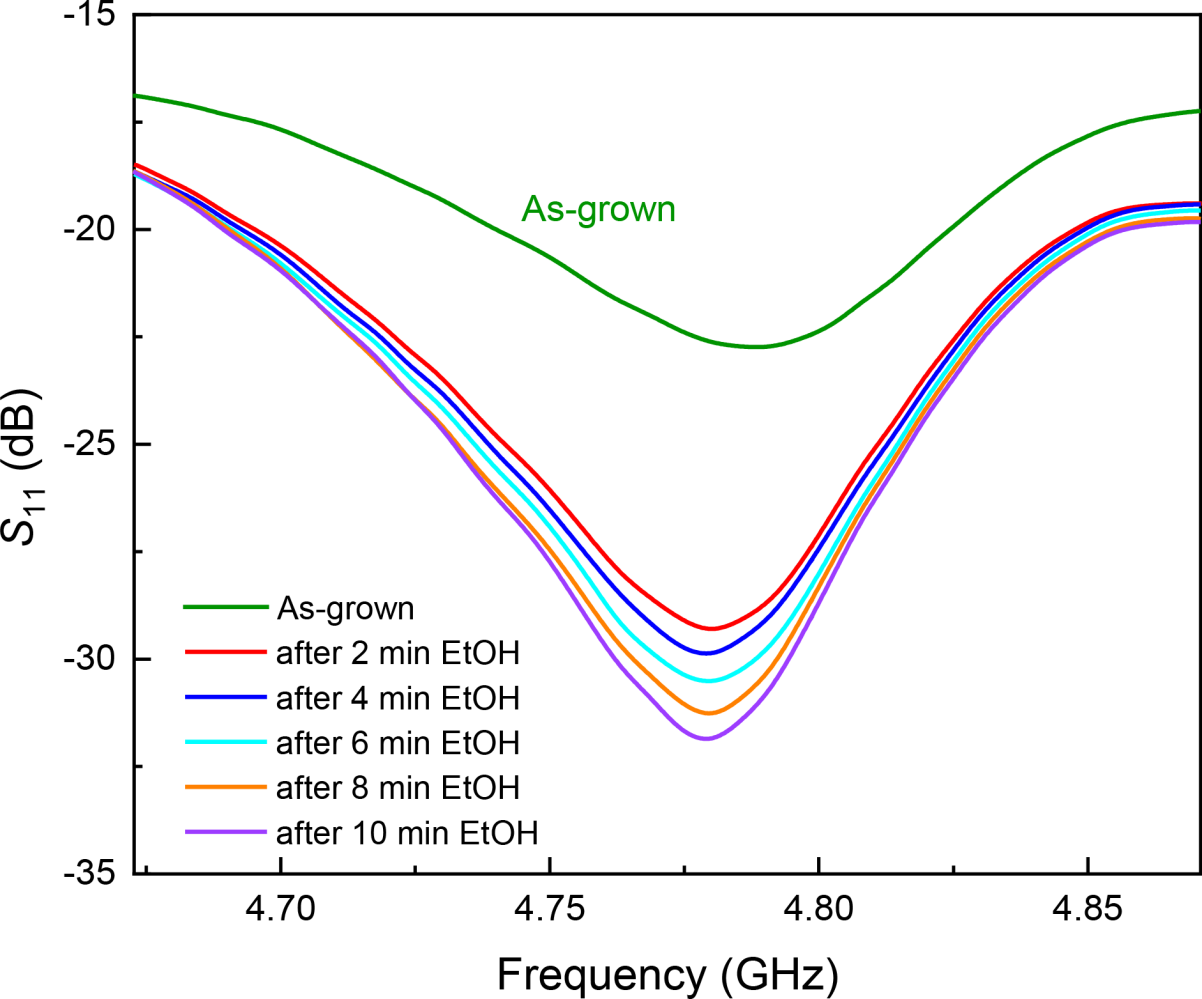
*S*_11_ reflection spectra collected on the as-grown CNS sample (green line) and acquired at different times after ethanol (EtOH) treatment (lines of different colours).

To increase the response of the CNS antenna, we measured the *S*_11_ response every 2 min after wetting the material with 5 μL of ethanol. Figure 2 reports the reflection spectra (*S*_11_) at the resonance peak found of the as-grown sample and after treatment with ethanol. It can be noticed that *S*_11_ was brought to a resonance peak at −29*.*4 dB after 2 min, and we observed a progressive improvement after waiting for some minutes after the treatment. The last *S*_11_ signal acquired after 10 min reached −32 dB.

This observed trend is similar to that reported by Xu et al. [29], which the authors explained on the basis of two distinct effects; one is the addition of scattering centres associated with the ethanol wetting, and the second is a densification of the material. While we agree with the first effect, which we think is not permanent because ethanol evaporates after some time, our findings point to a different interpretation related to the morphological effect of ethanol on the CNT mesh [27,29].

**Figure 3:**
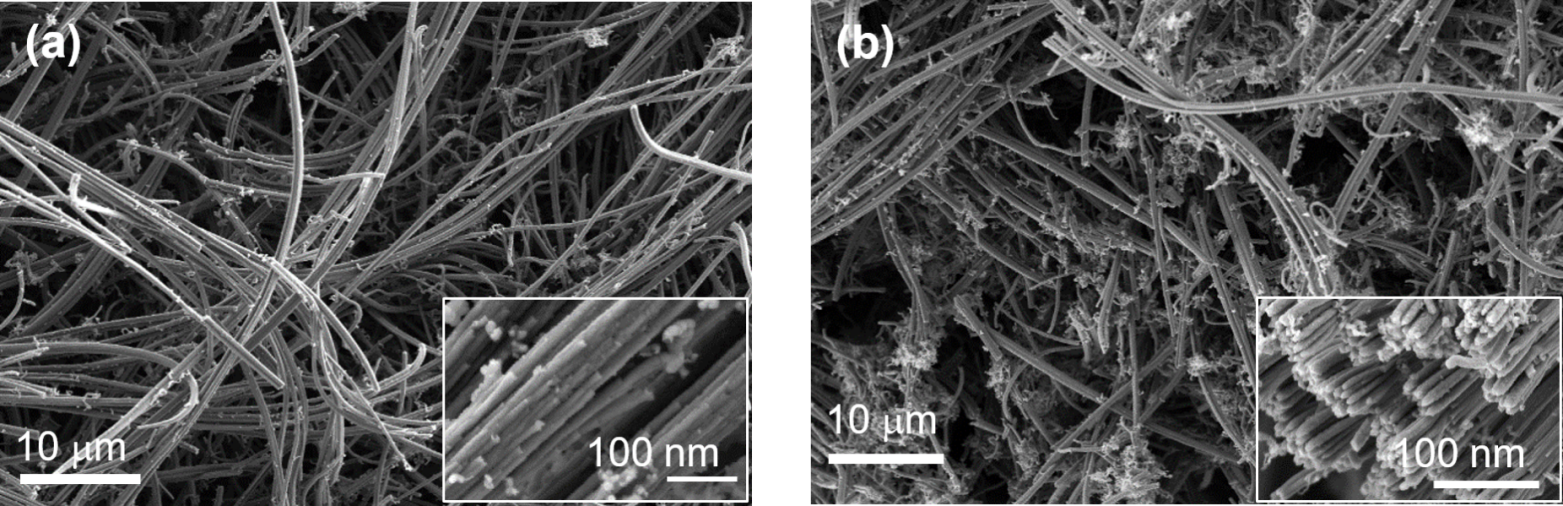
SEM micrographs of the CNS sample. (a) As-grown sample and (b) after ethanol treatment. Both images contain an inset with a magnified view of the sample. Scale bars are reported in the images.

A scanning electron microscopy (SEM) study provided insights into the microstructure of the CNT film (Supporting Information File 1, Figure S1) and the sponges (Figure 3). The study conducted on the as-grown sample revealed an interconnected network of MWCNTs arranged in bundles with external tube diameters ranging between 60 and 80 nm (Figure 3a) and a porosity above 95% [24–27]. After the treatment with ethanol, tubes of smaller diameters and lengths appear, and the bundles are separated, exposing the ends of the tubes (Figure 3b). This behaviour is comparable to that found in similar studies reported by Yu and coworkers [30].

**Figure 4:**
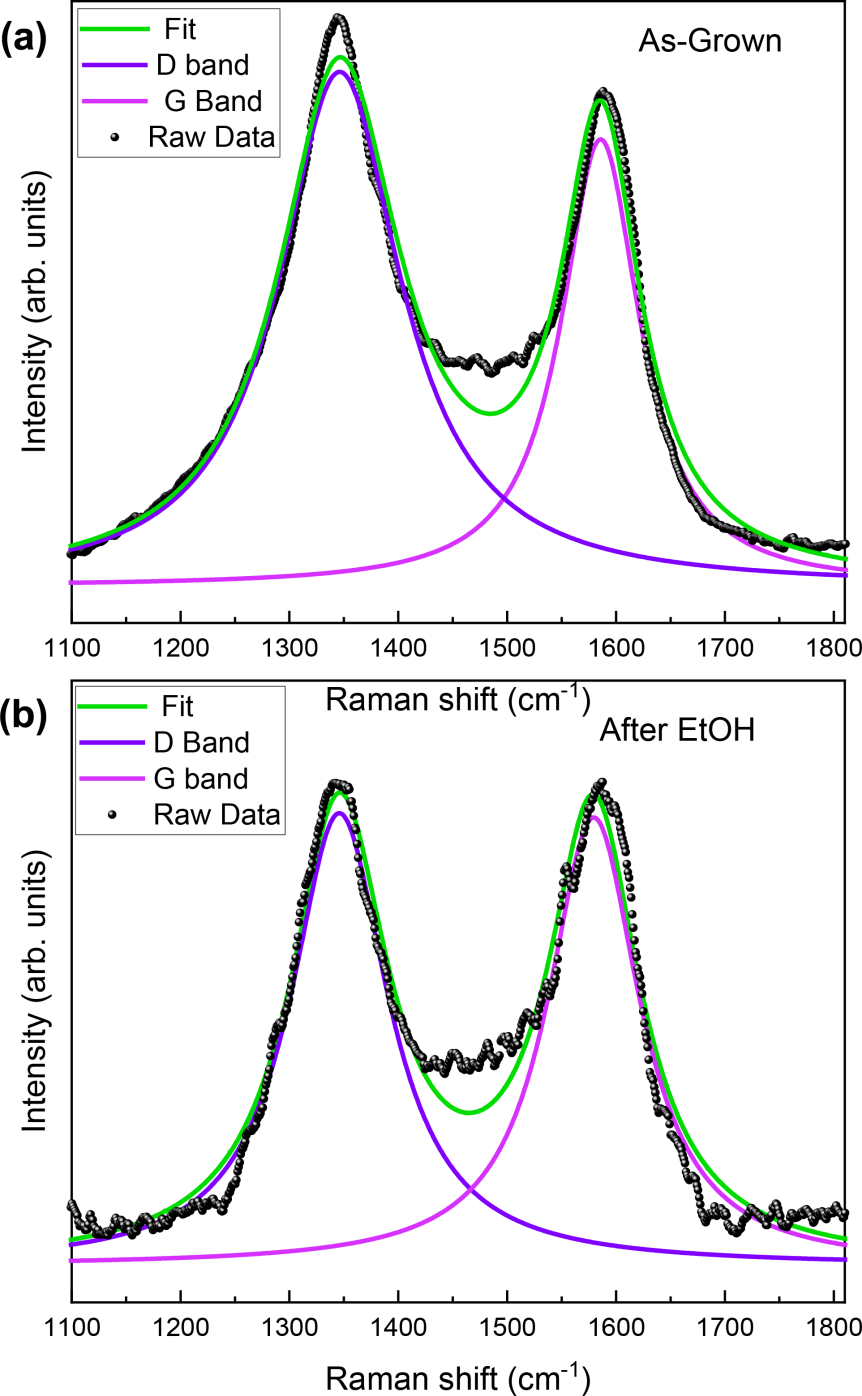
Raman spectra of the CNS sample (a) before and (b) after ethanol treatment.

To get a better insight into the effect of ethanol treatment, we acquired Raman spectra of the as-grown sample and 10 min after ethanol treatment (Figure 4). The spectral range studied was 1100–1800 cm^−1^, where we observed the contributions from the D and G bands. The D band is associated with the presence of defects and disorder in the sp^2^ lattice, while the G band is a fingerprint of the sp^2^ order [31]. The D band is located at 1345 ± 2 cm^−1^ and the G band at 1587 ± 2 cm^−1^. We did not observe any appreciable shift of these two components in the treated sample. By comparing the outcome of the fit procedure, we estimated from the area of the two peaks a reduction of the D band after the ethanol treatment of 20% ± 1% and a corresponding increase of the G band of 34% ± 1%. This trend is confirmed by the *I*_G_/*I*_D_ ratio, which increases from an initial value of 0.88 to 1.00. It must be noticed that the D band is already intense in the as-grown sample; it originates from the presence of defects in the tubes’ outer walls and from the highly interconnected structure of the samples. The Raman analysis results are in line with the morphological information obtained from the SEM data. The SEM and Raman experiments confirm the effect of ethanol on the CNS, which improves the presence of grooves and pores as available adsorption sites after ethanol treatment.

**Figure 5:**
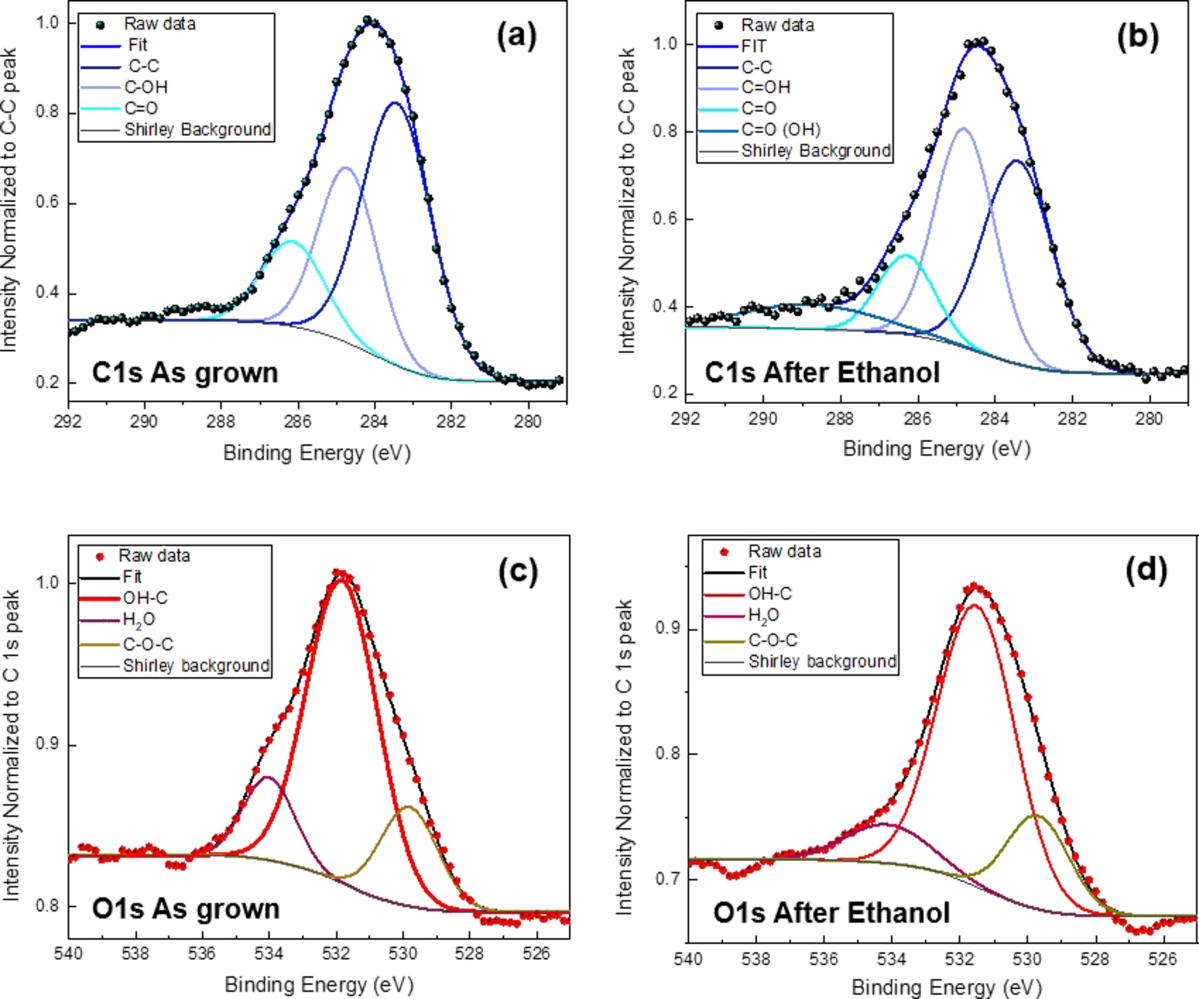
C 1s and O 1s core level spectra obtained from (a, c) the as-grown samples and (b, d) after ethanol treatment.

We also investigated the chemical state of the carbon atoms by acquiring X-ray photoelectron spectroscopy (XPS) spectra from the as-grown and ethanol-treated CNS samples. The survey spectra are reported in Supporting Information File 1 (Figure S2). Figure 5 shows that the C1 s spectra are composed mainly of electrons excited from the sp^2^ and sp^3^ hybridization of the honeycomb lattice and from the defects in the structure that also comprises OH groups. In particular, the spectra were fitted by the sum of three main components assigned to carbon–carbon bonds (C=C/C−C/C−H, 284.4 ± 0.1 eV), hydroxy groups (C−OH, 285.7 ± 0.1 eV), and carbonyl groups (C=O, 287.1 ± 0.1 eV) [32]. After treatment with ethanol, signals of all aforementioned oxygen-containing components increased in intensity, while that of the sp^2^ component decreased. Furthermore, carboxyl groups (C=O(OH), 290 ± 0.1 eV) were added as components to the spectra. In order to provide more definite results, an additional deconvolution of the O1 s core level spectra is presented (Figure 5c,d). The fits include the C 1s signals of most of the oxygen–carbon components (C–O–C, 529.8 ± 0.1 eV) and (OH–C, 531.8 ± 0.1 eV ). In the spectra of as-grown and ethanol-treated CNS samples, a small peak appears at 534 ± 0.1 eV, attributed to water that easily adheres to the tubes outer wall due to air exposure. Table S2 in Supporting Information File 1 gives a summary of the functional group content obtained from the C1 s and O1 s core level fits in Figure 5.

From the Raman and XPS studies, as well as the SEM micrographs, we conclude that the ethanol treatment induced a change in the structure of the CNS sample, unravelling many of the aggregated tubes, resulting in an increase in pore size and overall available surface area. Without losing the 3D structure, and as long as ethanol drops are present on the tubes, there is also an increase in interfacial reflection, similar to what is reported in [29], but the latter effect is not permanent. Therefore, we believe that the morphological effect is the important one.

After testing the capability of CNT film and CNS to work as antennas in the gigahertz regime, we carried out signal reception tests simulating real-world operation conditions to evaluate the practical application only for the CNS samples as RF antennas. The experimental setup consisted of a transmitting antenna (a dipole designed to resonate at the target frequency) and a receiving configuration incorporating a CNS antenna. A detailed description of the setup is reported in Supporting Information File 1 (Figure S3 and Figure S4). For the transmitting part, a simple, custom-made dipole antenna, appropriately sized at λ/4 was connected, which transmitted a 5 dBm signal at the resonant frequency of 4.8 GHz; also, a minor signal was observed at 5.2 GHz. The CNS antenna was placed at distances of (3.0 ± 0.1) × 10^−1^ m and (7.0 ± 0.1) × 10^-1^ m.

**Figure 6:**
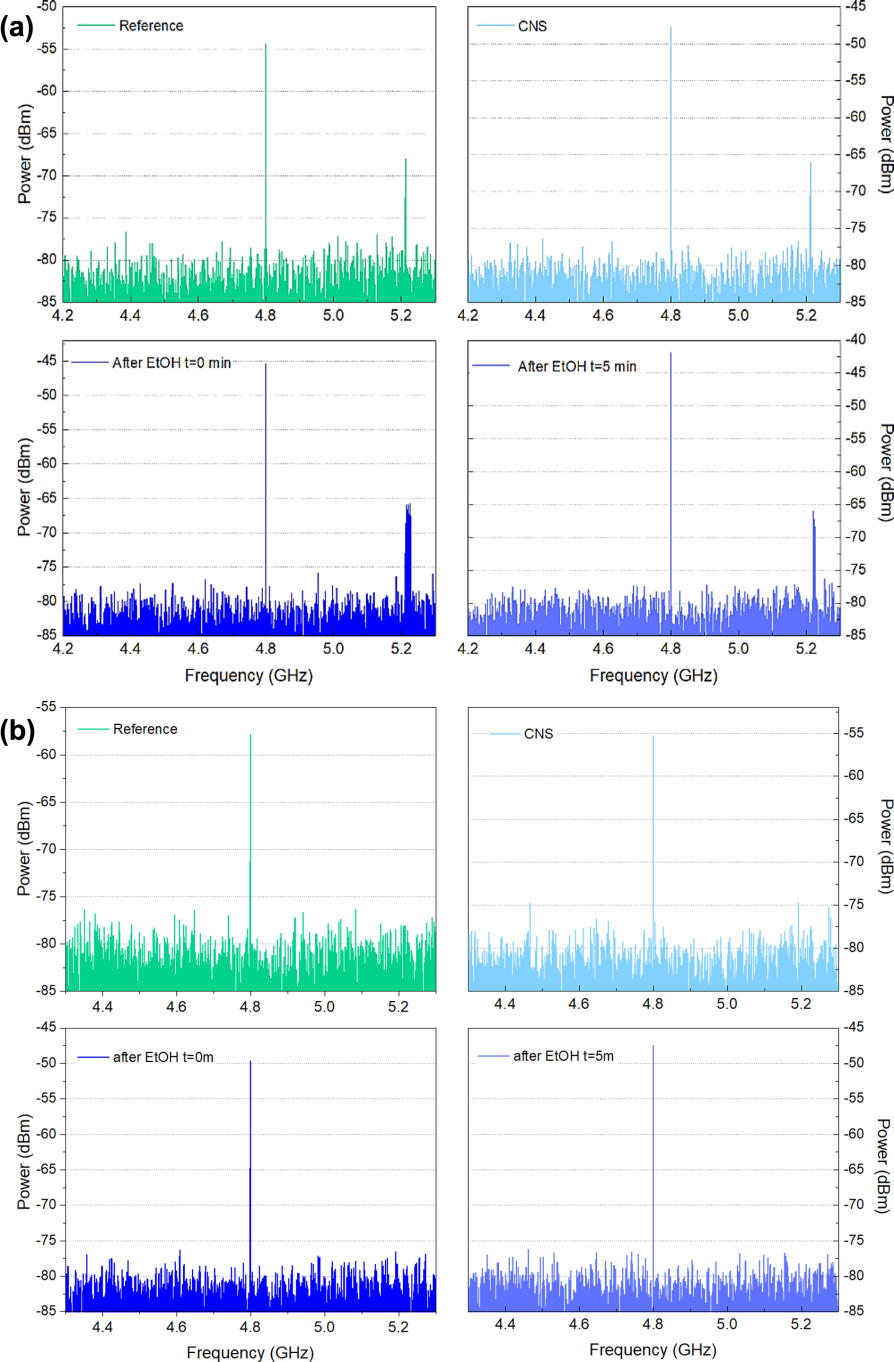
Results of the CNS as a signal receiver positioned (a) 30 cm and (b) 70 cm away from the emitting antenna. The reference signal is also reported.

Figure 6a reports the results over a distance of (3.0 ± 0.1) × 10^−1^ m in free space. The response registered from the untreated CNS shows a gain of 7 dBm; directly after ethanol treatment, the gain improved to 10 dBm, and to 13 dBm after 5 min. This result validates the *S*_11_-derived efficiency enhancements found. The trend was confirmed for the distance of (7.0 ± 0.1) × 10^−1^ m in free space (Figure 6b), for which we observed a gain of 2 dBm for the untreated sample, a 7 dBm gain directly after ethanol treatment, and a 10 dBm gain after 5 min.

The power density at 4.8 GHz was calculated for a resonant dipole antenna with a gain of 2.15 dB, accounting for a 0.7 dB cable loss before the antenna. The input power was 5 dBm, which is 3.16 × 10^−3^ W, and the power reaching the antenna terminals after loss was (2.69 ± 0. 01) × 10^−3^ W. Using the far-field relation, the power density at a distance of 70 cm was found to be (7.17 ± 0.01) × 10^−4^ W·m^−2^; at 30 cm, it was (3.90 ± 0.01) × 10^−3^ W·m^−2^.

It is essential to emphasise the frequency selectivity of the CNS antenna towards the interference signal at 5.2 GHz (Wi-Fi band, Figure 6a). For this frequency, no noticeable variation in the signal reception was observed with the CNS, highlighting potential applications in multiband RF systems as selective antenna that isolates signals at their resonant frequency (4.8 GHz). These results suggest that the highly electrically conductive CNS can be successfully utilized for the implementation of frequency-tunable and omnidirectional monopole patch RF antennas.

This experimental work demonstrates that CNSs offer practical advantages over CNT films since they operate as a self-standing material without the need of a substrate support. In particular, CNSs can absorb RF signals and offer an effective gain in terms of performance compared to a similar MWCNT film grown on a substrate. The ethanol treatment yielded excellent results, enhancing the efficiency of the CNS in absorbing RF signals and serving as a signal receptor. We explained the observed improvement by the structural modifications in the CNT assembly induced by ethanol. Compared with other doping strategies or external field excitation, our proposed method has the advantages of simplicity, ease of operation, wideband operation, and high modulation rate. This provides an idea for the research of tunable terahertz devices. As a signal receptor, we also observed a frequency selectivity in the CNS antenna towards the interference signal at 5.2 GHz that turned out to be isolated. This observed behaviour is essential in telecommunications to transmit information.

The CNS exhibited promising antenna characteristics, attributed to the high electrical conductivity of the MWCNTs, good impedance matching, and reduced electromagnetic interference due to the absence of a substrate. The 3D CNT structures can be produced through a facile low-cost synthesis process; due to the high flexibility, light weight, and good conductivity, they represent a novel material that can be exploited for integration into small devices and other applications demanding non-standard shapes that also need to resist thermal and mechanical stress or chemically harsh conditions.

## Experimental

The CNTs were synthesised using a chemical vapour deposition process [26–28] with a floating catalyst injection in a quartz tube (60 cm length and 45 mm external diameter) horizontally positioned inside a furnace (Lenton, UK). The CNT films were grown on a silicon substrate (n-doped) placed inside a quartz tube that was sealed and pumped down to 10^−2^ mbar to reduce contamination and remove oxygen. The temperature was then increased at a constant rate (5 °C·min^−1^) up to 750 °C. Before the growth process, the pressure was brought to 1 atm by introducing a mixture of argon and hydrogen (30 and 20 sccm, respectively) through a stainless steel pipe. A solution containing ferrocene (2.3 wt %) dispersed in 10 mL of ethanol and stirred for 4 h was injected at a constant rate (5 mL·h^−1^) into the pipe and carried inside the hot area of the furnace by the gas mixture with the addition of acetylene (20 sccm). In the hot area, the precursor dissociated, and the CNTs started growing. The process was stopped after 30 min. The system was then allowed to cool down to room temperature.

The synthesis of the CNSs was similar. The differences were: (1) No substrate was used. (2) Thiophene (1% v/v) was added to the solution as defect inducer [26–29] in addition to ferrocene (2.3 wt %), both dispersed in 10 mL of ethanol, stirred for 4 h and injected at a constant rate (5 mL·h^−1^) into the pipe during the growth process. (3) The pressure of the gas mixture (argon, acetylene, and hydrogen at 200, 80, and 100 sccm, respectively) was higher than that adopted for the growth of the CNT film. (4) The process duration was 2 h. After cooling down the system, the reaction products were directly collected from the quartz tube. Table S1 in Supporting Information File 1 details the growth parameters adopted for the CNT samples. The CNS bulk material was segmented to a size of 1.5 cm × 1.5 cm with a height of 0.4 cm for the experiment.

The treatment with ethanol consisted of wetting the CNS sample with 5 μL of ethanol (97.7–98.5% v/v, purity > 99.9%, Sigma-Aldrich Co.) and then acquiring the response every 2 min.

A field-emission scanning electron microscope Carl Zeiss Sigma 300 was used to collect images directly on the CNS samples and on the MWCNT films. Micro-Raman analysis was performed using a laser with a wavelength of 532 nm and a power of approximately 15 mW. The photon energy was calibrated using a Si reference. The measurements were performed using a monochromator with 1800 lines·mm^−1^. The laser power was optimised to prevent laser-induced damage.

XPS surface analysis studies were performed in an ultrahigh-vacuum chamber (base pressure below 10^−10^ bar) equipped with a semi-imaging analyser MAC 2 (Riber Instruments) operating in the constant pass energy mode (with a total energy resolution of 1.1 eV). Non-monochromatic Al Kα radiation (1486.6 eV) was used (8 kV, 8 mA). The CNS samples were fixed on a molybdenum sample holder with silver paint kept at a distance of about 40 mm from the anode, the illumination area was about 5 mm × 5 mm, and the take-off angle between the sample surface and the energy analyser was kept at 45°. Survey and high-resolution spectra were acquired. Typically, ten scans were accumulated for each acquired spectrum in three different regions of the sample. The XPS core level spectra were analysed using a fit routine, which decomposes each spectrum into separate Gaussian–Lorentzian peaks of variable widths after Shirley background subtraction. The quality of the fit was evaluated using a χ^2^ minimisation test. All binding energies were referenced to C 1s at around 284.4 eV, and the estimated errors in peak positions are of about ±0.1 eV.

The performance of the CNT sponges and the CNT film as RF antennas was evaluated by measuring their reflection coefficient (*S*_11_) using a vector network analyser from Agilent (E5071C ENA VNA) operating in the frequency range from 300 kHz to 20 GHz.

The transmitting system was driven by a Rohde & Schwarz SMR20 RF generator, while the received signal was analysed using an SM200B spectrum analyser.

## Supporting Information

File 1 Additional details on CNS preparation, XPS analysis, and antenna characterization.

## Data Availability

The data generated and analysed during this study is available from the corresponding author upon reasonable request.
