# Supplementary material for: Interconnection morphology effects on the radio frequency response of carbon nanotube sponges
Source: Beilstein J Nanotechnol. 2026 Feb 17;17:343–51. doi: 10.3762/bjnano.17.23 (PMC12927487; doi:10.3762/bjnano.17.23)
Supplement: File 1 — Additional details on CNS preparation, XPS analysis, and antenna characterization. [file Beilstein_J_Nanotechnol-17-343-s001.pdf]

BEILSTEIN JOURNAL OF NANOTECHNOLOGY

# Supporting Information

for

# Interconnection morphology effects on the radio frequency response of carbon nanotube sponges

Manuela Scarselli, Javad Rezvani, Zeno Zuccari, Mattia Scagliotti and Simone Tocci

*Beilstein J. Nanotechnol.* **2026,** *17,* 343–351. doi:10.3762/bjnano.17.23

## Additional details on CNS preparation, XPS analysis, and antenna characterization

License and Terms: This is a supporting information file under the terms of the Creative Commons Attribution License (https://creativecommons.org/licenses/by/4.0). Please note that the reuse, redistribution and reproduction in particular requires that the author(s) and source are credited and that individual graphics may be subject to special legal provisions.
The license is subject to the *Beilstein Journal of Nanotechnology* terms and conditions: (https://www.beilstein-journals.org/bjnano/terms)

# Synthesis of the carbon nanotube films and the 3D assembly

Figure S1 reports a SEM micrograph of the CNT film grown on the silicon substrate. In the inset is a magnified area of the film.

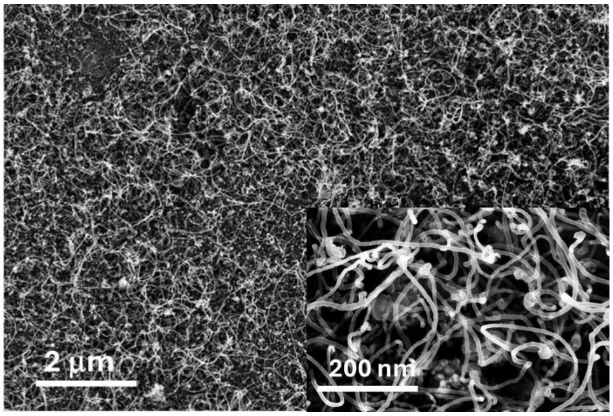


**Figure S1:** SEM micrograph of the CNT film grown on a silicon substrate.

Table S1 summarizes the parameters adopted for the synthesis of the studied CNT samples.

**Table S1:** CNT synthesis parameters: growth temperature (°C), gas partial pressure (sccm), and growth time (h).

| Sample | Growth temp. [°C] | $C_2H_2$ [sccm] | $H_2$ [sccm] | Ar [sccm] | Growth time [h] |
|---|---|---|---|---|---|
| CNT film on Si | 750 | 20 | 20 | 50 | 0.5 |
| CNT sponge | 750 | 80 | 100 | 200 | 2 |

# XPS measurements

Figure S2 reports an XPS survey spectrum obtained from the CNS sample before and after ethanol treatment, where the C 1s and O 1s core levels are highlighted. The ratios between the C 1s and O 1s signals in each spectrum are reported in the image; it can be observed that, after ethanol treatment, the ratio decreases due to an increase in oxygen content. Table 2 reports a summary of the functional group content obtained from the C 1s and O 1s core level fits of Figure 5 in the main manuscript.

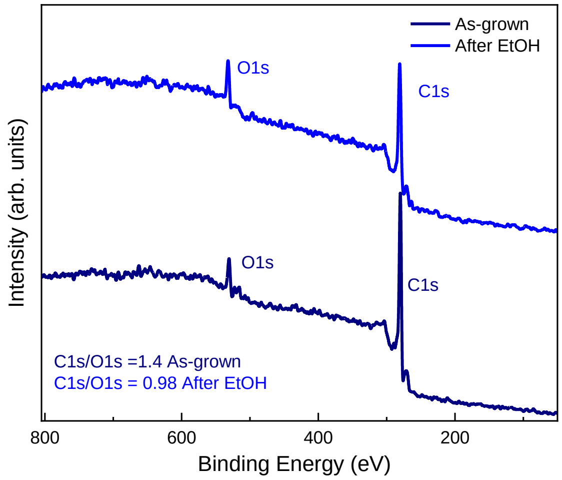


**Figure S2:** XPS survey spectra obtained on the as-grown CNS sample (dark blue line) and after ethanol treatment (light blue line).

**Table S2:** Functional group content obtained from the C 1s and O 1s core level fits.

| | Sample | C–C ($sp^2$) [%] | C–OH [%] | C=O [%] | C=O (OH) [%] |
|---|---|---|---|---|---|
| C 1s | as-grown | 54.0 | 30.3 | 15.7 | 0.0 |
| C 1s | after EtOH | 36.2 | 37.7 | 16.8 | 9.3 |
| | | C–O–H [%] | OH–C [%] | water [%] | |
| O 1s | as-grown | 18.0 | 68.6 | 13.4 | |
| O 1s | after EtOH | 19.0 | 70.4 | 10.6 | |

# Measurements on the CNT antennas

The setup used for the measurements includes an Agilent E5071C ENA vector network analyser and a coaxial cable (Figure S3). The Network analyser sends signals at frequencies between 300 kHz and 20 GHz and measures the reflected signal, generating an $S_{11}$ curve. A coaxial cable is connected to the instrument, and the samples to be analysed are mounted on the hot pole of the latter.

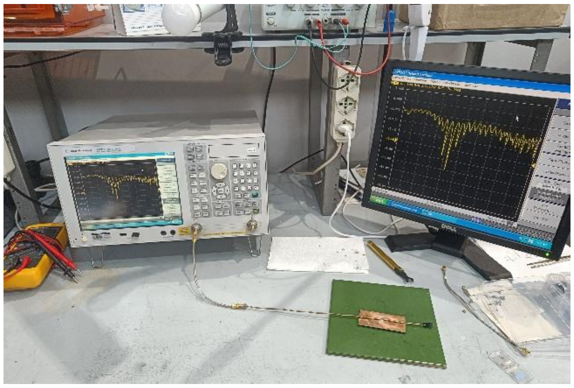

**Figure S3:** Experimental setup for the $S_{11}$ measurement.

Figure S4 shows the schematic of the dipole antenna built for the measurement. The antenna is composed of two straight, aligned conductors separated by a small gap, one connected to the hot pole and the other to the ground of a coaxial cable. The antenna is powered at the centre, where an alternating signal generates an oscillating electric field along the arms, enabling long-distance communication.

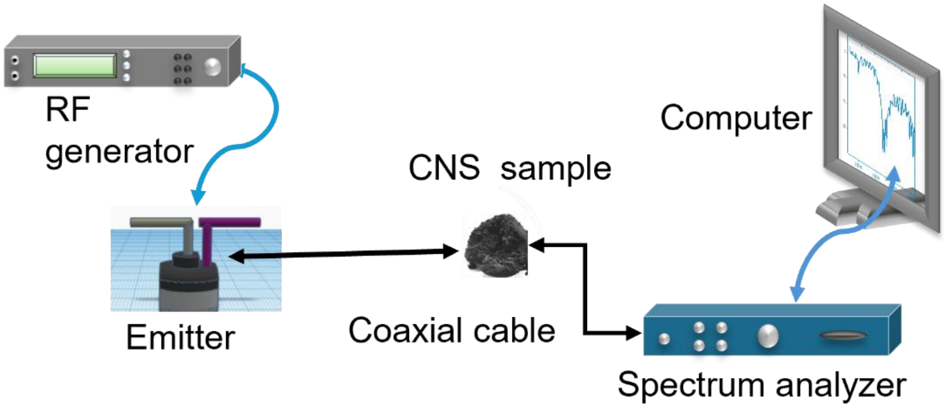


**Figure S4**: Schematics of the setup for the signal reception measurements.

Figure S5 shows a blow-up centred around the maximum value registered for the antenna at distances of $(3.0 \pm 0.1) \times 10^{-1}$ m and $(7.0 \pm 0.1) \times 10^{-1}$ m.

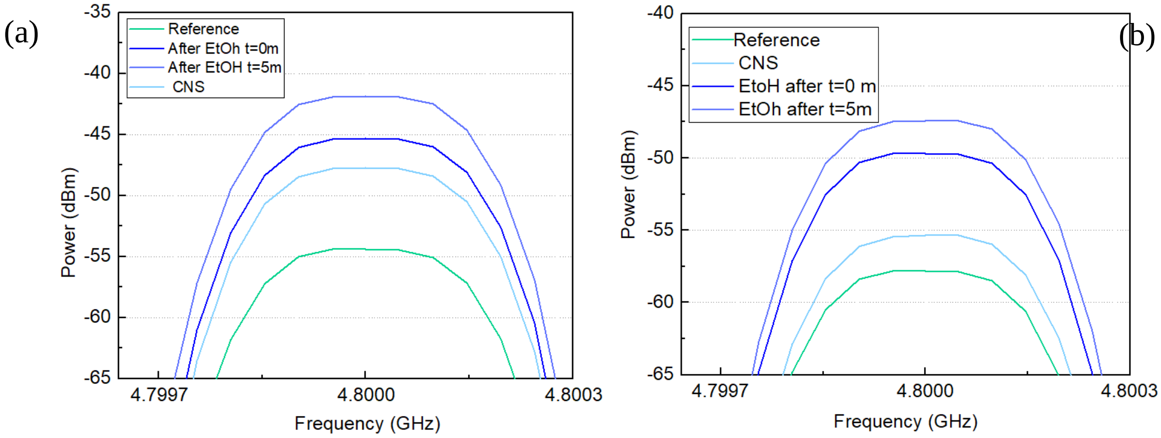


**Figure S5:** Enlarged view around the maximum value registered for the CNS antenna at distances of (a) $(3.0 \pm 0.1) \times 10^{-1}$ m and (b) $(7.0 \pm 0.1) \times 10^{-1}$ m.
